# Uncovering the Pharmacology of Xiaochaihu Decoction in the Treatment of Acute Pancreatitis Based on the Network Pharmacology

**DOI:** 10.1155/2021/6621682

**Published:** 2021-03-20

**Authors:** Lianghui Zhan, Jinbao Pu, Yijuan Hu, Pan Xu, Weiqing Liang, Chunlian Ji

**Affiliations:** ^1^Zhejiang Academy of Traditional Chinese Medicine, Hangzhou, Zhejiang 310014, China; ^2^Key Laboratory of Research and Development of Chinese Medicine of Zhejiang Province, Hangzhou, Zhejiang 310014, China; ^3^Tongde Hospital of Zhejiang Province, Hangzhou, Zhejiang 310014, China

## Abstract

**Background:**

Xiaochaihu decoction (XD) has demonstrated the pharmacodynamics on acute pancreatitis. This study was aimed at investigating the material and molecular basis of Xiaochaihu decoction.

**Methods:**

Firstly, compounds of seven herbs containing XD were collected from the TCMSP, ETCM, and BATMAN-TCM databases, and the putative targets of pancreatitis were obtained from the OMIM, TTD, and GeneCards databases. Then, the PPI network was constructed according to the matching results between XD potential targets and pancreatic neoplasm targets. Furthermore, enrichment analysis on GO and KEGG by DAVID utilized bioinformatics resources. Finally, molecular docking was performed to simulate the interaction between the active compound of XD and putative targets. In an *in vitro* experiment, AR42J cells were induced by LPS and then treated with Quercetin (25, 50, and 100 *μ*M) or XCHD. The IL-6, TNF-*α*, and IL-1*β* levels were detected by ELISA kit, *MAPK3* and *TP53* mRNA expressions were measured by qRT-PCR, and the proteins of MAPK3 and TP53 expressions were measured by WB.

**Results:**

A total of 196 active ingredients and 91 putative targets were selected. The PPI network analysis demonstrated that Quercetin was the candidate agent and MAPK3, IL-6, and TP53 were the potential targets for the XD treatment of acute pancreatitis. The KEGG analysis revealed that pathways in cancers, TNF signaling way, and MAPK signaling way might play an important role in pancreatitis therapy. And molecular docking results showed that Quercetin combined well with MAPK3, IL-6, and TP53. An *in vitro* experiment indicated that XCHD and Quercetin inhibited the IL-6, TNF-*α*, and IL-1*β* levels and MAPK3 and TP53.

**Conclusion:**

This study illustrated that XCHD and Quercetin contained in XD played an important role in the treatment of acute pancreatitis by acting on the key genes of MPAK3, IL-6, and TP53 which were associated with inflammation and apoptosis.

## 1. Introduction

Acute pancreatitis is a highly variable disease characterized by acute inflammation and necrosis of the pancreatic parenchyma which is associated with a high mortality of about 20%-30% [1–3]. And it brought on mainly by some factors such as gallstones, chronic alcohol, and obesity [4–6]. Acute pancreatitis could divide into local complications including ascites and acute fluid collection, as well as infected necrosis and systemic complications including single organ failure or multiple organ dysfunction syndrome (MODS) [7, 8]. At present, the major therapeutic measures for acute pancreatitis are symptomatic treatments such as easement of pain and the correction of fluid, electrolyte, and pH balances [9]. Currently, there is lack of effective therapeutic strategy for acute pancreatitis, so valid drugs need to be developed. Due to the widespread application of Traditional Chinese Medicine (TCM), it has been testified that Chinese decoctions had significant curative effect on the treatment of acute pancreatitis [10, 11].

Xiaochaihu decoction (XD) was chronicled in Shanghan Lun, a famous Chinese ancient book, which is composed of Chaihu (*Radix Bupleuri*), Banxia (*Arum ternatum Thunb*), Renshen (*Panax ginseng C. A. Mey*.), Gancao (*licorice*), Huangqin (*Scutellariae Radix*), Shengjiang (*Zingiber officinale Roscoe*), and Dazao (*Jujubae Fructus*), and in recent years, experiments showed that XD was beneficial to prevention and cure of acute pancreatitis [12, 13]. Zhang et al. indicated that XD could protect the pancreas against chronic injury and improve pancreatic exocrine function in a DBTC-induced rat CP model [14]. This hinted that XD might be a potential alternative medicine for the treatment of acute pancreatitis, but its pharmacological mechanism is not well understood.

With the rapid development of network pharmacology in systems biology, it is frequently used to systematically investigate the interaction between Chinese medicine and the complicated human body [15]. Network pharmacology was combined with systems biology, pharmacology, and computer technology to explore the complex mechanism by which Chinese formulations treat complex diseases [16]. Furthermore, network pharmacology applied to the research of TCM could analyze the rationality of the pharmacodynamics mechanism [17].

In the study, we aimed to use a comprehensive network pharmacology-based approach to investigate the mechanisms of how XD exerts the therapeutic effects on acute pancreatitis. Further, the potential mechanism in an *in vitro* experiment was also verified.

## 2. Materials and Methods

### 2.1. Data Collection of 7 Herbs Contained in XD

We collected the chemical ingredients of 7 herbs contained in the Xiaochaihu decoction by the TCMSP (Traditional Chinese Medicine Systems Pharmacology, https://tcmspw.com/tcmsp.php), ETCM (the Encyclopedia of Traditional Chinese Medicine, http://www.tcmip.cn/ETCM/index.php/Home/), and BATMAN-TCM (http://bionet.ncpsb.org/batman-tcm/) databases [18–20]. Then, the requested ingredients were screened according to the conditions of oral bioavailability (OB) ≥ 30% and drug likeness (DL) ≥ 0.18. The putative targets of 7 herbs contained in the Xiaochaihu decoction were searched in the DrugBank database (https://www.drugbank.ca/unearth/advanced/bio_entities).

### 2.2. The Putative Targets of Acute Pancreatitis Collection

By using “acute pancreatitis” in order to search words, we searched out the putative targets in the GeneCards database (https://auth.lifemapsc.com/), OMIM database (https://www.omim.org/), and TTD database (http://db.idrblab.net/ttd/) with the species limited as “Homo sapiens.” Then, we removed the duplicate value to get the relative putative value.

### 2.3. Gene Name Correction and Common Target Screening

Firstly, the gene names of the Xiaochaihu decoction and acute pancreatitis were adjusted by the UniProt database (https://www.uniprot.org/) and then the intersection map of component targets and disease targets was made by a Venn map to obtain the intersection targets and further to get the potential therapeutic targets of XD on the treatment of acute pancreatitis.

### 2.4. TCM-Compound-Target-Disease Network Construction

Intersection targets obtained from the Venn map were reverse screened for corresponding chemical ingredients and herbs. And then the TCM-compound-target-disease network could be constructed which was also visualized by Cytoscape 3.6.1.

### 2.5. Protein-Protein Interaction (PPI) Network

Targets obtained from the Venn map were uploaded to the STRING database (http://string-db.org/), and the PPI network was generated with the species limited as “Homo sapiens” and medium confidence as “0.4.” And the acquired PPI network was imported into Cytoscape 3.6.1 to visually analyze.

### 2.6. Pathway Enrichment Performance

The intersection targets were imported into the DAVID database (https://david.ncifcrf.gov/) and then the gene function was obtained as well as the effects in the pathway. Gene Ontology (GO) and pathway enrichment analyses were performed, setting the list type to gene list and limiting the species to Homo sapiens, and the top 20 terms were sorted to draw a histogram by GraphPad Prism. The Kyoto Encyclopedia of Genes and Genomes database (KEGG, http://www.genome.jp/kegg/) analysis was visualized by the ggplot2 database.

### 2.7. Molecular Docking Method

Active compounds owed the most targets, and targets closely related with acute pancreatitis were imported into Discovery Studio 4 software, and then molecular docking was performed by using the CDOCKER model.

### 2.8. Experiment Validation

#### 2.8.1. Cell Culture

Rat pancreatic acinar AR42J cells were obtained from the China Center for Type Culture Collection (CCTCC, China). AR42J cells were maintained in 1640 RPMI medium containing 10% FBS and 100 U/mL penicillin and plated at the density of 1 × 10^5^ cells/mL in a 96-well culture plate at 37°C and 5% CO_2_.

#### 2.8.2. Drug-Contained Serum Preparation

Xiaochaihu decoction was composed of Chaihu (24 g), Banxia (9 g), Huangqin (9 g), Shengjiang (9 g), Renshen (9 g), Gancao (9 g), and Dazao (12 g) which were mixed and concentrated to the certain dose (2 g/mL). And male SD rats (200 ± 20 g) were administrated with XD for 3 days, and the blood was collected from the rat eye at the last day. And the blood was centrifuged to get serum and stored in -80°C.

#### 2.8.3. MTT Assay

AR42J cells in each group were treated with LPS (Sigma, America) of various concentrations 1, 2, and 4 *μ*g/mL for 24 hours to investigate the best condition for the subsequent experiment. Quercetin (Sigma, America) at the concentrations of 12.5, 25, 50, and 100 *μ*M and serum containing Xiaochaihu decoction (XCHD) were incubated with AR42J cells for 8 hours after LPS treated. The cells were washed with PBS for 3 times after removing the culture solution and incubated with 5 mg/mL MTT at 37°C for 4 hours. And then, 150 *μ*L DMSO was added to stop the reaction, and the reaction liquid was detected at 570 nm. Each experiment was repeated for 3 times.

#### 2.8.4. The Determination of IL-6, TNF-*α*, and IL-1*β*

After being treated with LPS and XCHD or Quercetin, AR42J cells in the cell culture dishes of each group were trypsinized and collected by centrifugation at 37°C for 5 min at a speed of 2500 r/min. The factor interleukin-6 (IL-6), tumor necrosis factor *α* (TNF-*α*), and interleukin-1 (IL-1*β*) were detected by ELISA kit (09/2019, Shanghai Enzyme-linked Biotechnology Co., Ltd.) according to the protocol provided by the manufacturer.

#### 2.8.5. TP53 and MAPK3 mRNA Expression Detected by qRT-PCR

After treated with LPS for 24 hours and Quercetin for another 8 hours, the total RNA of AR42J cells were isolated by TRIzol methods (Invitrogen, CA, USA). The integrity was detected by 1% agarose gel electrophoresis (AGE), and the concentrations of total RNA and OD260/OD280 value were measured by a microspectrophotometer. The procedure of reverse transcription of total RNA was performed in strict accordance with the manufacturer's instructions (CWBIO, Beijing, China). Then, the relative expressions of *TP53* and *MAPK3* mRNA were detected by qRT-PCR. The PCR primers were designed based on the NCBI reference sequence database for each gene and using Primer 5 software (Table 1), and the expansion conditions were as follows: heating to 95°C for 10 min, 40 cycles of 5°C for 15 sec, 55°C for 30 sec, and 72°C for 30 sec were applied [19]. GAPDH was used as a reference gene, and comparison in the expression between groups was made using the 2-*ΔΔ*CT method.

#### 2.8.6. The Protein of P53 and MAPK3 Expression Detected by Western Blot

After being treated with LPS for 24 hours and XCHD for another 8 hours, the total protein of AR42J cells was extracted by radioimmunoprecipitation lysis buffer (RIPA, Beyotime, Shanghai, China). And then, the protein was separated by 10% SDS PAGE to the PVGF membrane. The membrane was blocked with skim milk (5%) for 2 hours before being incubated with the antibody of MAPK3 (1 : 200, Proteintech, USA), TP53 (1 : 200, Proteintech, USA), and *β*-actin (1 : 1000, Proteintech, USA) overnight at 4°C. On the second day, the membrane was washed with PBS for 3 times and then incubated with HRP-IgG (1 : 5000, Proteintech, USA) for 2 hours. Finally, the membrane was visualized with an enhanced chemiluminescence (ECL) detection system. The density of each band was estimated using the Image Lab software. All target proteins were normalized against the loading control *β*-actin.

### 2.9. Data and Statistical Analysis

Statistical analysis was processed with IBM SPSS Statistics 19.0 (SPSS Inc., NY, USA). Data were expressed as the mean ± SD and analyzed using Student's *t*-test. Differences between groups were considered to be statistically significant if values of *P* < 0.05.

## 3. Results

### 3.1. Active Ingredients of Xiaochaihu Decoction

There were 196 active ingredients of 7 herbs contained in the Xiaochaihu decoction which were collected from the TCMSP Database with the limited lists “OB ≥ 30%” and “OL ≥ 0.18.” As is shown in Table S1, the active composition included 17 in Chaihu, 13 in Banxia, 36 in Huangqin, 22 in Renshen, 29 in Dazao, 5 in Shengjiang, and 94 in Gancao.

### 3.2. Intersection Targets of Xiaochaihu Decoction and Acute Pancreatitis

**Figure 1:**
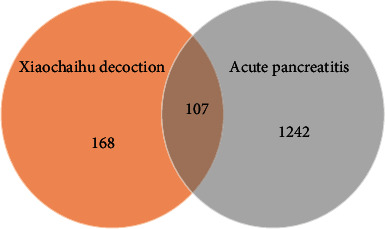
Venn map of common targets on Xiaochaihu decoction and acute pancreatitis. The orange circle indicates the targets of Xiaochaihu decoction, and the gray circle indicates the targets of acute pancreatitis. The part of the two intersecting circles is the common targets.

The Xiaochaihu decoction had 168 targets obtained from the DrugBank database, and acute pancreatitis had 1242 targets collected from the OMIM, GeneCards, and TTD databases. Then, the Venn map showed that it had 107 intersection targets on the Xiaochaihu decoction and acute pancreatitis (Figure 1).

### 3.3. Herb-Compound-Target-Disease Network Analysis

**Figure 2:**
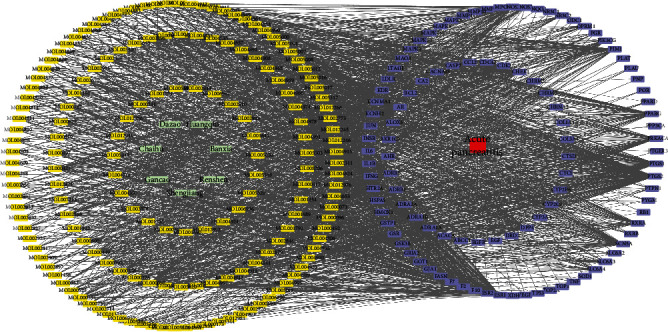
Herb-compound-target-disease network. The green squares indicate the herb name, the blue squares indicate the compounds, the purple squares indicate the target, and the red square indicates the disease name.

To clarify the relationship between the herbs of the Xiaochaihu decoction, the compound target, and acute pancreatitis targets, the herb-compound-target-disease network was constructed by Cytoscape software. The network had 279 nodes consisting of 1 disease, 7 herbs, 164 compounds, and 107 putative targets. In terms of the relationship between compounds and targets, there were 12 compounds having targets greater than 30, and they were MOL000098 (Quercetin), MOL000422 (Kaempferol), MOL003896 (7-Methoxy-2-methyl isoflavone), MOL000787 (Fumarine), MOL000354 (Isorhamnetin), MOL000392 (Formononetin), MOL002565 (Medicarpin), MOL000358 (Beta-sitosterol), MOL000449 (Stigmasterol), MOL004978(2-[(3R)-8,8-Dimethyl-3,4-dihydro-2H-pyrano[6,5-f]chromen-3-yl]-5-methoxyphenol), MOL000500 (Vestitol), and MOL004891 (Shinpterocarpin) acting on 75, 44, 43, 38, 34, 34, 34, 32, 31, 31, 30, and 30 targets, respectively. And PTGS2, HSP90A, CAMKK2, ESR1, AR, PTGS1, NOS2, NCOA2, PRSS1, F10, and SCN5A interacted with 138, 100, 97, 90, 89, 86, 83, 74, 73, and 71 compounds (Figure 2).

### 3.4. PPI Network Analysis

**Figure 3:**
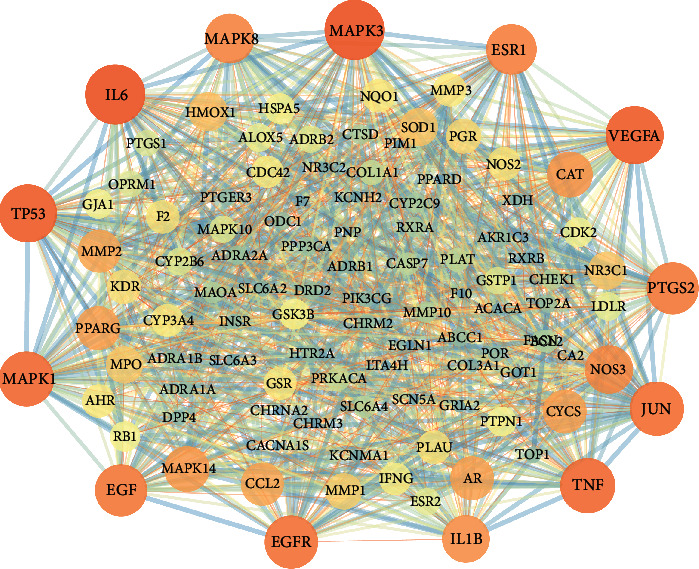
Protein-protein interaction network (PPI network). Orange, yellow, and green circles indicated the proteins. The lines represented the nodes associated through protein-protein interactions.

The common targets of compounds and acute pancreatitis were put into the STRING database to obtain the PPI network in order to provide an intuitive understanding of the mechanism of the Xiaochaihu decoction acting on acute pancreatitis. By analysis of STRING, it was shown that the network was composed with 108 nodes as well as 1143 edges, and the average node degree was 21.2. Furthermore, network topological analysis indicated that the top 3 degrees were mitogen-activated protein kinase 3 (MAPK3, degree = 62), interleukin-6 (IL-6, degree = 62), and tumor protein (TP53, degree = 59), which were involved with the cell growth and cell apoptosis as well as inflammatory response, and had the greater node degree value than the other targets (Figure 3**)**.

### 3.5. Enrichment of Function Analysis

**Figure 4:**
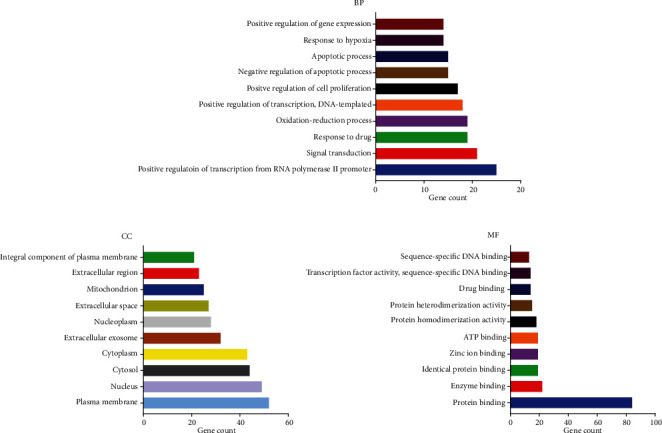
Gene Ontology (GO) enrichment analysis of the top 20 pathways: (a) biological process (BP); (b) cell component (CC); (c) molecular function (MF).

To clarify the mechanism of the Xiaochaihu decoction on acute pancreatitis in detail, the GO enrichment analysis was performed by the DAVID Bioinformatics Resources. GO enrichment analysis got 251 items including 139 items of the biological process (BP), 62 items of the cell component (CC), and 50 items of the molecular function (MF) (*P* < 0.05), and the items with the gene count proportion greater than 10 are shown in Figure 4. The biological process was mainly related to positive regulation of transcription from RNA polymerase II promoter, and molecular function was closely connected with protein binding.

**Figure 5:**
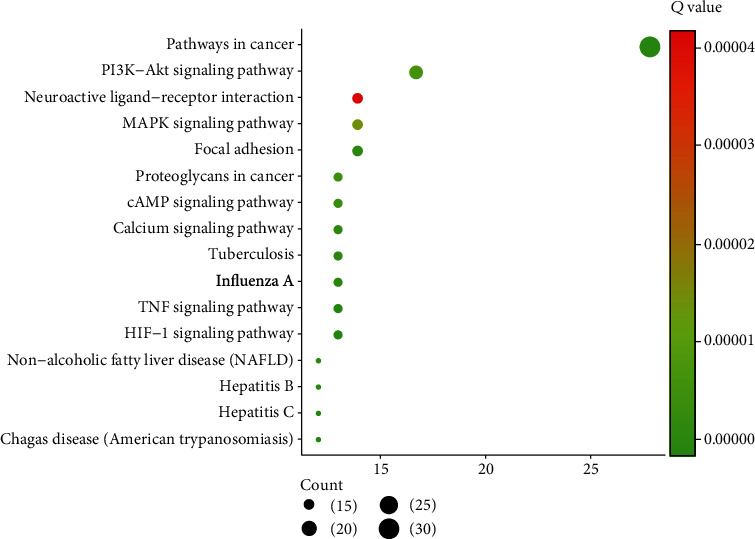
Kyoto Encyclopedia of Genes and Genomes (KEGG) pathway enrichment. The color of circles represents the *Q* value, and the size of circles represents the count.

KEGG pathway analysis indicated that the Xiaochaihu decoction which exerted its pharmacological effects on acute pancreatitis was closely associated with pathways in cancers (fold enrichment = 5.4, *P* < 0.001), TNF signaling way (fold enrichment = 12.2, *P* < 0.001), PI3K-Akt signaling pathway (fold enrichment = 3.7, *P* < 0.001), influenza A (fold enrichment = 7.1, *P* < 0.001), Chagas disease (fold enrichment = 11.0, *P* < 0.001), and MAPK signaling pathway (fold enrichment = 4.5, *P* < 0.001) which were involved with MAPK3, TP53, TNF, and so on (Figure 5).

### 3.6. Molecular Docking to Determine the Potential Targets

**Figure 6:**
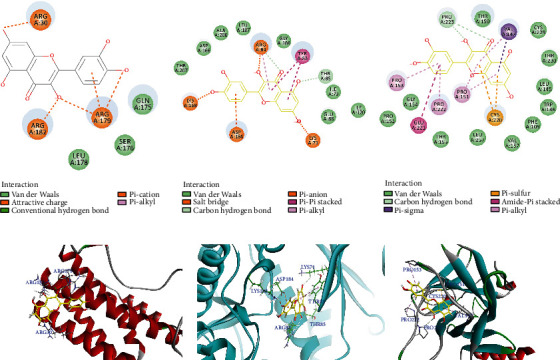
The protein-ligand of the docking simulation: (a) Quercetin and IL-6 (2D); (b) Quercetin and MAPK3 (2D); (c) Quercetin and TP53 (2D); (d) Quercetin and IL-6 (3D); (e) Quercetin and MAPK3 (3D); (f) Quercetin and TP53 (3D).

Three targets (IL-6, MAPK3, and TP53) were selected according to the results of the PPI network as the core targets of the Xiaochaihu decoction treated acute pancreatitis. And herb-compound-target-disease network indicated that Quercetin not only could interact with 75 targets but also was closely related with the three targets. Therefore, molecular docking technology was used to simulate the interaction between Quercetin and the three targets. The results showed that Quercetin interacted with IL-6, MAPK3, and TP53, forming van der Waals, carbon hydrogen bond, Pi-sulfur, salt bridge, and so on (Figure 6).

### 3.7. XCHD and Quercetin Increased the AR42J Cell Viability

**Figure 7:**
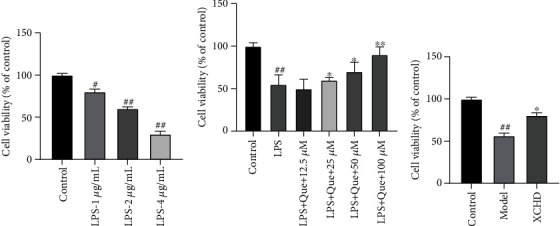
The effect of LPS, Quercetin, and XCHD on AR42J cell viability. (a) AR42J cells were treated with LPS at 1, 2, and 4 *μ*g/mL for 24 hours. (b) AR42J cells were incubated with Quercetin (12.5, 25, 50, and 100 *μ*M) for 8 hours after being treated with 1 *μ*g/mL LPS. (c) AR42J cells were incubated with XCHD-contained serum for 8 hours after being treated with 1 *μ*g/mL LPS. Data are expressed as the mean ± SD of three independent experiments. ^#^*P* < 0.05 and ^##^*P* < 0.01 compared with the control group; ^∗^*P* < 0.05 and ^∗∗^*P* < 0.01 compared with the model group.

To investigate the best concentrations of LPS to build an acute pancreatitis model *in vitro*, AR42J cell viability was measured following treatment of 1, 2, and 4 *μ*g/mL LPS for 24 hours. As is shown in Figure 7(a), AR42J cell viability was achieved to nearly 60% with the treatment of LPS at the dose of 2 mg/mL for 24 hours which was for the subsequent experiment.

According to the results of herb-compound-target-disease network analysis, Quercetin was the mainly active compound of Xiaochaihu decoction. Quercetin (12.5, 25, 50, and 100 *μ*M) was treated with AR42J cells for 8 hours after AR42J cells were incubated with LPS (2 mg/mL) for 24 hours to detect cell viability by MTT assay. It was shown that Quercetin at the concentrations of 25, 50, and 100 *μ*M significantly improved the cell viability (*P* < 0.05, 0.01), but Quercetin at a dose of 12.5 *μ*M had less effect on it (Figure 7(b)).

And then Xiaochaihu decoction-contained serum (XCHD) was treated with AR42J cells for 8 hours after AR42J cells were incubated with LPS (2 mg/mL) for 24 hours to detect cell viability by MTT assay. It was shown that XCHD improved the cell viability significantly which was reduced by LPS treatment, compared with the model group (*P* < 0.05). (Figure 7(c)).

### 3.8. Quercetin and XCHD Inhibited the Production of IL-6, IL-1*β*, and TNF-*α*

**Figure 8:**
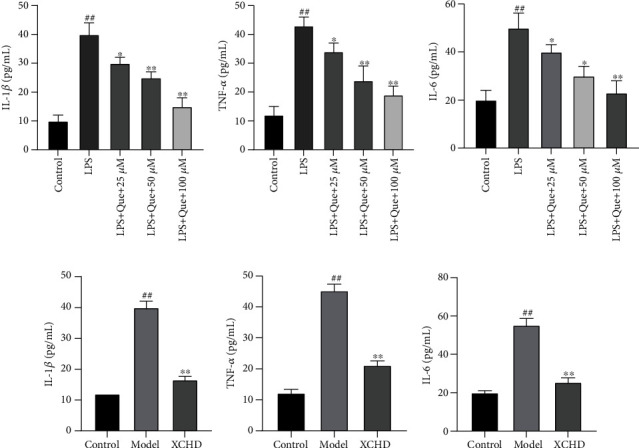
The effects of Quercetin and XCHD on the production of IL-6, IL-1*β*, and TNF-*α* in LPS-induced acute pancreatitis *in vitro*. After LPS (2 mg/mL) was treated with AR42J cells for 24 hours, Quercetin (a–c) or XCHD (d–f) was incubated with the cells for another 8 hours. And the production of IL-1*β* (a, d), IL-6 (b, e), and TNF-*α* (c, f) was detected by ELISA kit. Data are expressed as the mean ± SD of three independent experiments. ^#^*P* < 0.05 and ^##^*P* < 0.01 compared with the control group; ^∗^*P* < 0.05 and ^∗∗^*P* < 0.01 compared with the model group.

According to the PPI network analysis, Xiaochaihu decoction was closely association with inflammatory reaction. Therefore, the levels of IL-6, IL-1*β*, and TNF-*α* in LPS-induced pancreatitis model *in vitro* were detected. LPS significantly increased the secretion of IL-1*β*, IL-6, and TNF-*α* compared with control group. As expected, Quercetin at 25, 50, and 100 *μ*M reduced the generation of these inflammatory factors (*P* < 0.05, 0.01, Figures 8(a)–8(c)).

Besides, it also investigated the effect of XCHD on the generation of IL-6, IL-1*β*, and TNF-*α* in an LPS-induced acute pancreatitis model. As shown in Figures 8(d)–8(f), LPS obviously increased the production of IL-6, IL-1*β*, and TNF-*α* (*P* < 0.01). However, XCHD retarded the surging tendency of IL-6, IL-1*β*, and TNF-*α* as expected (*P* < 0.01).

### 3.9. Quercetin and XCHD Downregulated the Expression of MAPK3 and TP53

**Figure 9:**
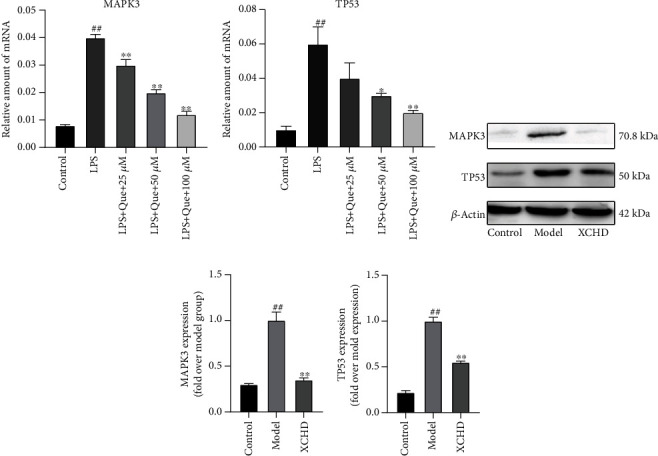
Changes in expressions of MAPK3 and TP53 in AR42J cells following LPS, Quercetin, and (or) XCHD treatment. (a) Real-time PCR analysis of *MAPK3* in AR42J cells. (b) Real-time PCR analysis of *TP53* in AR42J cells. (c) WB analysis of MAPK3 and TP53 in AR42J cells. (d, e) The statistics on MPAK3 and TP53. Data are expressed as the mean ± SD of three independent experiments. ^#^*P* < 0.05 and ^##^*P* < 0.01 compared with the control group; ^∗^*P* < 0.05 and ^∗∗^*P* < 0.01 compare with the model group.

The PPI network and KEGG results all showed that Xiaochaihu decoction functioning in the treatment of insomnia was involved with MAPK3 and TP53 which was further confirmed by molecular docking technology. Firstly, it can be seen in Figures 9(a) and 9(b), Quercetin obviously inhibited the relative expression of *MAPK3* and *TP53* mRNA which were upregulated after LPS treatment (*P* < 0.01). Besides, In the *in vitro* experiment (Figures 9(c)–9(e)), the proteins of MAPK3 and TP53 were both upregulated after AR42J cells treated with LPS significantly (*P* < 0.01). However, compared with the model group, XCHD obviously reduced the relative expressions MAPK3 and TP53 (*P* < 0.01).

## 4. Discussion

Xiaochaihu decoction is an ancient herbal formula which has therapeutic agents for the treatment of acute pancreatitis in the clinic [21]. In this study, a network pharmacology approach was applied to construct the “herb-compound-target-disease network” in order to explore the underlying mechanism of Xiaochaihu decoction in acute pancreatitis. Results of the study described herein revealed that the Xiaochaihu decoction was exerted on the acute pancreatitis by multiple pathways and target points.

Acute pancreatitis was an inflammation condition, and some cytokines could be predictors and markers of disease severity [22]. A clinical reported indicated that during the early acute pancreatitis stage, pancreatitis acinar cells always produced the cytokines such as tumor necrosis factor *α* (TNF-*α*) and interleukin-1*β* (IL-1*β*) [23]. Besides, the injured pancreatic acinar cells from acute pancreatitis patients also had the initial inflammatory responses to expressed cytokines such as actor interleukin-6 (IL-6) [24]. In other words, inflammation played an important role in the development of acute pancreatitis [25]. Stojanovic et al. also demonstrated that acute pancreatitis could be attenuated by inhibiting the activation of innate inflammatory cells [26]. The Xiaochaihu decoction had the effect of regulating qi-flowing for strengthening the spleen and promoting circulation and removing stasis in TCM which could conform to the therapeutic theory of acute pancreatitis [27]. Furthermore, it was also reported that the Xiaochaihu decoction could reduce the IL-1*β* and other proinflammatory factors to improve inflammatory disease [28]. As expected, in this study, XCHD indeed improved the viability of AR42J cells which triggered inflammatory damage by LPS. And then, according to the results of the network pharmacology analysis, Quercetin (OB = 46.43%, DL = 0.28) became the potential bioactive compounds. As is well-known, Quercetin inhibited the LPS-induced TNF-*α*, IL-1*β*, and IL-6 production in several studies *in vitro* [29, 30]. Junyuan et al. also proved that Quercetin protected against inflammation in acute necrotizing pancreatitis [31]. And in the present study, Quercetin as the predicted compound of the Xiaochaihu Decoction showed that significantly anti-inflammation ability by suppressing the IL-6, TNF-*α*, and IL-1*β* levels.

PPI network analysis also showed that the degree of TP53 (degree = 59) and MAPK3 (degree = 62) were higher than the others. TP53, the regulatory factor of apoptosis, was usually expressed at the low level under normal physical condition, but could be accumulated rapidly after cell DNA damage [32, 33]. Therefore, in the clinic, pancreatic tissues from patients with acute pancreatitis always exhibited apoptotic nuclei and increased p53 expression [34]. Consistently, Zhou et al. found that TP53 suppressed on a mouse acute pancreatitis model obviously inhibited pancreatic acinar cell apoptosis and the inflammation [35]. Also, the expression of TP53 was obviously decreased after the treatment of XCHD and Quercetin in the current study. And mitogen-activated protein kinase 3 (MAPK3) was an essential component of the MAPK signal transduction pathway which mediated diverse biological functions such as cell growth, adhesion, survival, and differentiation through the regulation of transcription, translation, and cytoskeletal rearrangements [36]. And KEGG analysis revealed that the MAPK signaling pathway had linkage with the Xiaochaihu decoction treatment for acute pancreatitis. And it was also demonstrated that MPAK3 was functionally related to the migration of the inflammation cell [37]. Kim et al. found that the severity of acute pancreatitis could be attenuated by inhibited proinflammatory cytokine production and acinar cell death via inhibiting the activation of MPAK3 [38]. Therefore, the study showed that the expression of MAPK3 was obviously decreased after XCHD and Quercetin treatment. Taken together, the results indicated that TP53 and MAPK3 might be a putative target of the Xiaochaihu decoction treated with acute pancreatitis.

## 5. Conclusion

In general, In general, this study demonstrated that Xiaochaihu decoction treated with acute pancreatitis by acting the key genes of MAPK3, IL-6 and TP53 using network pharmacology method and molecular docking technology. Furthermore, it was demonstrated that XCHD and Quercetin reduced the inflammation and apoptosis factor (IL-1*β*, IL-6, TNF-*α*, MAPK3, and TP53) generation in the *in vitro* experiment. However, the pharmacodynamics effect of the Xiaochaihu decoction treated on acute pancreatitis was needed to validated *in vivo*, and the further mechanism still remains to be systematically explored.

## Figures and Tables

**Table 1:** PCR primers.

Gene	Sequence
*TP53*	F:5′-TCAGCATCTTATCCGAGTGGAA-3′ R:5′-AGGGCACCACCACACTATGTC-3′
*MAPK3*	F:5′-AACCCAAACAAGCGCATCAC-3′ R:5′-TCGGATCGTAGTACTGTTCCAGGTA-3′
*GAPDH*	F:5′-CATGAGAAGTATGACAACAGCCT-3′ R:5′-AGTCCTTCCACGATACCAAAGT-3′

GADPH was used as an internal control. TP53: tumor protein 53; MAPK3: mitogen-activated protein kinase 3.

## Data Availability

All data generated or analyzed during this study are included in this published article.

## Abbreviations

XD: Xiaochaihu decoction
XCHD: Serum containing Xiaochiahu decoction
TCM: Traditional Chinese medicine
TCMSP: Traditional Chinese Medicine Systems Pharmacology
BP: Biological process
CC: Cell component
MF: Molecular function
GO: Gene Ontology
KEGG: Kyoto Encyclopedia of Genes and Genomes
IL-1*β*: Interleukin-1*β*
IL-6: Factor interleukin-6
TNF-*α*: Tumor necrosis factor *α*.

## Supplementary Materials

Supplementary Materials Supplementary Table S1: the active ingredients of Xiaochaihu decoction.
