# Supplementary material for: Uncovering the Pharmacology of Xiaochaihu Decoction in the Treatment of Acute Pancreatitis Based on the Network Pharmacology
Source: Biomed Res Int. 2021 Mar 20;2021:6621682. doi: 10.1155/2021/6621682 (PMC8007340; doi:10.1155/2021/6621682)
Supplement: Supplementary Materials — Supplementary Table S1: the active ingredients of Xiaochaihu decoction. [file 6621682.f1.doc]

Table.S1 Active ingredients information of Xiaochaihu Decoction

| Mol ID | Molecule Name | OB (%) | DL | Herb |
| --- | --- | --- | --- | --- |
| MOL000073 | ent-Epicatechin | 48.96 | 0.24 | *Scutellariae Radix* |
| MOL000096 | (-)-catechin | 49.68 | 0.24 | *Jujubae Fructus* |
| MOL000098 | quercetin | 46.43 | 0.28 | *Radix Bupleuri、licorice、Jujubae Fructus* |
| MOL000173 | wogonin | 30.68 | 0.23 | *Scutellariae Radix* |
| MOL000211 | Mairin | 55.38 | 0.78 | *licorice、Jujubae Fructus* |
| MOL000228 | (2R)-7-hydroxy-5-methoxy-2-phenylchroman-4-one | 55.23 | 0.2 | *Scutellariae Radix* |
| MOL000239 | Jaranol | 50.83 | 0.29 | *licorice* |
| MOL000354 | isorhamnetin | 49.6 | 0.31 | *Radix Bupleuri、licorice* |
| MOL000358 | beta-sitosterol | 36.91 | 0.75 | *Arum Ternatum Thunb、Panax ginseng C. A. Mey.、Scutellariae Radix、Zingiber officinale Roscoe、Jujubae Fructus* |
| MOL000359 | sitosterol | 36.91 | 0.75 | *Scutellariae Radix、licorice* |
| MOL000392 | formononetin | 69.67 | 0.21 | *licorice* |
| MOL000417 | Calycosin | 47.75 | 0.24 | *licorice* |
| MOL000422 | kaempferol | 41.88 | 0.24 | *Radix Bupleuri、Panax ginseng C. A. Mey.、licorice* |
| MOL000449 | Stigmasterol | 43.83 | 0.76 | *Arum Ternatum Thunb、Panax ginseng C. A. Mey.、Scutellariae Radix、Zingiber officinale Roscoe、Jujubae Fructus、Radix Bupleuri* |
| MOL000490 | petunidin | 30.05 | 0.31 | *Radix Bupleuri* |
| MOL000492 | (+)-catechin | 54.83 | 0.24 | *Jujubae Fructus* |
| MOL000497 | licochalcone a | 40.79 | 0.29 | *licorice* |
| MOL000500 | licochalcone a | 40.79 | 0.29 | *licorice* |
| MOL000500 | Vestitol | 74.66 | 0.21 | *licorice* |
| MOL000519 | coniferin | 31.11 | 0.32 | *Arum Ternatum Thunb* |
| MOL000525 | Norwogonin | 39.4 | 0.21 | *Scutellariae Radix* |
| MOL000552 | 5,2'-Dihydroxy-6,7,8-trimethoxyflavone | 31.71 | 0.35 | *Scutellariae Radix* |
| MOL000627 | Stepholidine | 33.11 | 0.54 | *Jujubae Fructus* |
| MOL000783 | Protoporphyrin | 30.86 | 0.56 | *Jujubae Fructus* |
| MOL000787 | Fumarine | 59.26 | 0.83 | *Panax ginseng C. A. Mey.、Jujubae Fructus* |
| MOL001454 | berberine | 36.86 | 0.78 | *Jujubae Fructus* |
| MOL001458 | coptisine | 30.67 | 0.86 | *Scutellariae Radix* |
| MOL001484 | Inermine | 75.18 | 0.54 | *licorice* |
| MOL001490 | bis[(2S)-2-ethylhexyl] benzene-1,2-dicarboxylate | 43.59 | 0.35 | *Scutellariae Radix* |
| MOL001506 | Supraene | 33.55 | 0.42 | *Scutellariae Radix* |
| MOL001522 | (S)-Coclaurine | 42.35 | 0.24 | *Jujubae Fructus* |
| MOL001645 | Linoleyl acetate | 42.1 | 0.2 | *Radix Bupleuri* |
| MOL001689 | acacetin | 34.97 | 0.24 | *Scutellariae Radix* |
| MOL001755 | 24-Ethylcholest-4-en-3-one | 36.08 | 0.76 | *Arum Ternatum Thunb* |
| MOL001771 | poriferast-5-en-3beta-ol | 36.91 | 0.75 | *Zingiber officinale Roscoe* |
| MOL001792 | DFV | 32.76 | 0.18 | *licorice* |
| MOL002311 | Glycyrol | 90.78 | 0.67 | *licorice* |
| MOL002565 | Medicarpin | 49.22 | 0.34 | *licorice* |
| MOL002670 | Cavidine | 35.64 | 0.81 | *Arum Ternatum Thunb* |
| MOL002714 | baicalein | 33.52 | 0.21 | *Arum Ternatum Thunb、Scutellariae Radix* |
| MOL002773 | beta-carotene | 37.18 | 0.58 | *Jujubae Fructus* |
| MOL002776 | Baicalin | 40.12 | 0.75 | *Arum Ternatum Thunb、Radix Bupleuri* |
| MOL002844 | Pinocembrin | 64.72 | 0.18 | *licorice* |
| MOL002879 | Diop | 43.59 | 0.39 | *Panax ginseng C. A. Mey.、Scutellariae Radix* |
| MOL002897 | epiberberine | 43.09 | 0.78 | *Scutellariae Radix* |
| MOL002908 | 5,8,2'-Trihydroxy-7-methoxyflavone | 37.01 | 0.27 | *Scutellariae Radix* |
| MOL002909 | 5,7,2,5-tetrahydroxy-8,6-dimethoxyflavone | 33.82 | 0.45 | *Scutellariae Radix* |
| MOL002910 | Carthamidin | 41.15 | 0.24 | *Scutellariae Radix* |
| MOL002911 | 2,6,2',4'-tetrahydroxy-6'-methoxychaleone | 69.04 | 0.22 | *Scutellariae Radix* |
| MOL002913 | Dihydrobaicalin_qt | 40.04 | 0.21 | *Scutellariae Radix* |
| MOL002914 | Eriodyctiol (flavanone) | 41.35 | 0.24 | *Scutellariae Radix* |
| MOL002915 | Salvigenin | 49.07 | 0.33 | *Scutellariae Radix* |
| MOL002917 | 5,2',6'-Trihydroxy-7,8-dimethoxyflavone | 45.05 | 0.33 | *Scutellariae Radix* |
| MOL002925 | 5,7,2',6'-Tetrahydroxyflavone | 37.01 | 0.24 | *Scutellariae Radix* |
| MOL002926 | dihydrooroxylin A | 38.72 | 0.23 | *Scutellariae Radix* |
| MOL002927 | Skullcapflavone II | 69.51 | 0.44 | *Scutellariae Radix* |
| MOL002928 | oroxylin a | 41.37 | 0.23 | *Scutellariae Radix* |
| MOL002932 | Panicolin | 76.26 | 0.29 | *Scutellariae Radix* |
| MOL002933 | 5,7,4'-Trihydroxy-8-methoxyflavone | 36.56 | 0.27 | *Scutellariae Radix* |
| MOL002934 | NEOBAICALEIN | 104.34 | 0.44 | *Scutellariae Radix* |
| MOL002937 | DIHYDROOROXYLIN | 66.06 | 0.23 | *Scutellariae Radix* |
| MOL003410 | Ziziphin_qt | 66.95 | 0.62 | *Jujubae Fructus* |
| MOL003578 | Cycloartenol | 38.69 | 0.78 | *Arum Ternatum Thunb* |
| MOL003648 | Inermin | 65.83 | 0.54 | *Panax ginseng C. A. Mey.* |
| MOL003656 | Lupiwighteone | 51.64 | 0.37 | *licorice* |
| MOL003896 | 7-Methoxy-2-methyl isoflavone | 42.56 | 0.2 | *licorice* |
| MOL004328 | naringenin | 59.29 | 0.21 | *licorice* |
| MOL004350 | Ruvoside_qt | 36.12 | 0.76 | *Jujubae Fructus* |
| MOL004492 | Chrysanthemaxanthin | 38.72 | 0.58 | *Panax ginseng C. A. Mey.* |
| MOL004598 | 3,5,6,7-tetramethoxy-2-(3,4,5-trimethoxyphenyl)chromone | 31.97 | 0.59 | *Radix Bupleuri* |
| MOL004609 | Areapillin | 48.96 | 0.41 | *Radix Bupleuri* |
| MOL004624 | Longikaurin A | 47.72 | 0.53 | *Radix Bupleuri* |
| MOL004628 | Octalupine | 47.82 | 0.28 | *Radix Bupleuri* |
| MOL004644 | Sainfuran | 79.91 | 0.23 | *Radix Bupleuri* |
| MOL004648 | Troxerutin | 31.6 | 0.28 | *Radix Bupleuri* |
| MOL004653 | (+)-Anomalin | 46.06 | 0.66 | *Radix Bupleuri* |
| MOL004702 | saikosaponin c_qt | 30.5 | 0.63 | *Radix Bupleuri* |
| MOL004718 | α-spinasterol | 42.98 | 0.76 | *Radix Bupleuri* |
| MOL004805 | (2S)-2-[4-hydroxy-3-(3-methylbut-2-enyl)phenyl]-8,8-dimethyl-2,3-dihydropyrano[2,3-f]chromen-4-one | 31.79 | 0.72 | *licorice* |
| MOL004806 | euchrenone | 30.29 | 0.57 | *licorice* |
| MOL004808 | glyasperin B | 65.22 | 0.44 | *licorice* |
| MOL004810 | glyasperin F | 75.84 | 0.54 | *licorice* |
| MOL004811 | Glyasperin C | 45.56 | 0.4 | *licorice* |
| MOL004814 | Isotrifoliol | 31.94 | 0.42 | *licorice* |
| MOL004815 | (E)-1-(2,4-dihydroxyphenyl)-3-(2,2-dimethylchromen-6-yl)prop-2-en-1-one | 39.62 | 0.35 | *licorice* |
| MOL004820 | kanzonols W | 50.48 | 0.52 | *licorice* |
| MOL004824 | (2S)-6-(2,4-dihydroxyphenyl)-2-(2-hydroxypropan-2-yl)-4-methoxy-2,3-dihydrofuro[3,2-g]chromen-7-one | 60.25 | 0.63 | *licorice* |
| MOL004827 | Semilicoisoflavone B | 48.78 | 0.55 | *licorice* |
| MOL004828 | Glepidotin A | 44.72 | 0.35 | *licorice* |
| MOL004829 | Glepidotin B | 64.46 | 0.34 | *licorice* |
| MOL004833 | Phaseolinisoflavan | 32.01 | 0.45 | *licorice* |
| MOL004835 | Glypallichalcone | 61.6 | 0.19 | *licorice* |
| MOL004838 | 8-(6-hydroxy-2-benzofuranyl)-2,2-dimethyl-5-chromenol | 58.44 | 0.38 | *licorice* |
| MOL004841 | Licochalcone B | 76.76 | 0.19 | *licorice* |
| MOL004848 | licochalcone G | 49.25 | 0.32 | *licorice* |
| MOL004849 | 3-(2,4-dihydroxyphenyl)-8-(1,1-dimethylprop-2-enyl)-7-hydroxy-5-methoxy-coumarin | 59.62 | 0.43 | *licorice* |
| MOL004855 | Licoricone | 63.58 | 0.47 | *licorice* |
| MOL004856 | Gancaonin A | 51.08 | 0.4 | *licorice* |
| MOL004857 | Gancaonin B | 48.79 | 0.45 | *licorice* |
| MOL004860 | licorice glycoside E | 32.89 | 0.27 | *licorice* |
| MOL004863 | 3-(3,4-dihydroxyphenyl)-5,7-dihydroxy-8-(3-methylbut-2-enyl)chromone | 66.37 | 0.41 | *licorice* |
| MOL004864 | 5,7-dihydroxy-3-(4-methoxyphenyl)-8-(3-methylbut-2-enyl)chromone | 30.49 | 0.41 | *licorice* |
| MOL004866 | 2-(3,4-dihydroxyphenyl)-5,7-dihydroxy-6-(3-methylbut-2-enyl)chromone | 44.15 | 0.41 | *licorice* |
| MOL004879 | Glycyrin | 52.61 | 0.47 | *licorice* |
| MOL004882 | Licocoumarone | 33.21 | 0.36 | *licorice* |
| MOL004883 | Licoisoflavone | 41.61 | 0.42 | *licorice* |
| MOL004884 | Licoisoflavone B | 38.93 | 0.55 | *licorice* |
| MOL004885 | licoisoflavanone | 52.47 | 0.54 | *licorice* |
| MOL004891 | shinpterocarpin | 80.3 | 0.73 | *licorice* |
| MOL004898 | (E)-3-[3,4-dihydroxy-5-(3-methylbut-2-enyl)phenyl]-1-(2,4-dihydroxyphenyl)prop-2-en-1-one | 46.27 | 0.31 | *licorice* |
| MOL004903 | liquiritin | 65.69 | 0.74 | *licorice* |
| MOL004904 | licopyranocoumarin | 80.36 | 0.65 | *licorice* |
| MOL004905 | 3,22-Dihydroxy-11-oxo-delta(12)-oleanene-27-alpha-methoxycarbonyl-29-oic acid | 34.32 | 0.55 | *licorice* |
| MOL004907 | Glyzaglabrin | 61.07 | 0.35 | *licorice* |
| MOL004908 | Glabridin | 53.25 | 0.47 | *licorice* |
| MOL004910 | Glabranin | 52.9 | 0.31 | *licorice* |
| MOL004911 | Glabrene | 46.27 | 0.44 | *licorice* |
| MOL004912 | Glabrone | 52.51 | 0.5 | *licorice* |
| MOL004913 | 1,3-dihydroxy-9-methoxy-6-benzofurano[3,2-c]chromenone | 48.14 | 0.43 | *licorice* |
| MOL004914 | 1,3-dihydroxy-8,9-dimethoxy-6-benzofurano[3,2-c]chromenone | 62.9 | 0.53 | *licorice* |
| MOL004915 | Eurycarpin A | 43.28 | 0.37 | *licorice* |
| MOL004917 | glycyroside | 37.25 | 0.79 | *licorice* |
| MOL004924 | (-)-Medicocarpin | 40.99 | 0.95 | *licorice* |
| MOL004935 | Sigmoidin-B | 34.88 | 0.41 | *licorice* |
| MOL004941 | (2R)-7-hydroxy-2-(4-hydroxyphenyl)chroman-4-one | 71.12 | 0.18 | *licorice* |
| MOL004945 | (2S)-7-hydroxy-2-(4-hydroxyphenyl)-8-(3-methylbut-2-enyl)chroman-4-one | 36.57 | 0.32 | *licorice* |
| MOL004948 | Isoglycyrol | 44.7 | 0.84 | *licorice* |
| MOL004949 | Isolicoflavonol | 45.17 | 0.42 | *licorice* |
| MOL004957 | HMO | 38.37 | 0.21 | *licorice* |
| MOL004959 | 1-Methoxyphaseollidin | 69.98 | 0.64 | *licorice* |
| MOL004961 | Quercetin der. | 46.45 | 0.33 | *licorice* |
| MOL004966 | 3'-Hydroxy-4'-O-Methylglabridin | 43.71 | 0.57 | *licorice* |
| MOL004974 | 3'-Methoxyglabridin | 46.16 | 0.57 | *licorice* |
| MOL004978 | 2-[(3R)-8,8-dimethyl-3,4-dihydro-2H-pyrano[6,5-f]chromen-3-yl]-5-methoxyphenol | 36.21 | 0.52 | *licorice* |
| MOL004980 | Inflacoumarin A | 39.71 | 0.33 | *licorice* |
| MOL004985 | icos-5-enoic acid | 30.7 | 0.2 | *licorice* |
| MOL004988 | Kanzonol F | 32.47 | 0.89 | *licorice* |
| MOL004989 | 6-prenylated eriodictyol | 39.22 | 0.41 | *licorice* |
| MOL004990 | 7,2',4'-trihydroxy－5-methoxy-3－arylcoumarin | 83.71 | 0.27 | *licorice* |
| MOL004991 | 7-Acetoxy-2-methylisoflavone | 38.92 | 0.26 | *licorice* |
| MOL004993 | 8-prenylated eriodictyol | 53.79 | 0.4 | *licorice* |
| MOL004996 | gadelaidic acid | 30.7 | 0.2 | *licorice* |
| MOL005000 | Gancaonin G | 60.44 | 0.39 | *licorice* |
| MOL005001 | Gancaonin H | 50.1 | 0.78 | *licorice* |
| MOL005003 | Licoagrocarpin | 58.81 | 0.58 | *licorice* |
| MOL005007 | Glyasperins M | 72.67 | 0.59 | *licorice* |
| MOL005008 | Glycyrrhiza flavonol A | 41.28 | 0.6 | *licorice* |
| MOL005012 | Licoagroisoflavone | 57.28 | 0.49 | *licorice* |
| MOL005013 | 18α-hydroxyglycyrrhetic acid | 41.16 | 0.71 | *licorice* |
| MOL005016 | Odoratin | 49.95 | 0.3 | *licorice* |
| MOL005017 | Phaseol | 78.77 | 0.58 | *licorice* |
| MOL005018 | Xambioona | 54.85 | 0.87 | *licorice* |
| MOL005020 | dehydroglyasperins C | 53.82 | 0.37 | *licorice* |
| MOL005030 | gondoic acid | 30.7 | 0.2 | *Arum Ternatum Thunb* |
| MOL005308 | Aposiopolamine | 66.65 | 0.22 | *Panax ginseng C. A. Mey.* |
| MOL005314 | Celabenzine | 101.88 | 0.49 | *Panax ginseng C. A. Mey.* |
| MOL005317 | Deoxyharringtonine | 39.27 | 0.81 | *Panax ginseng C. A. Mey.* |
| MOL005318 | Dianthramine | 40.45 | 0.2 | *Panax ginseng C. A. Mey.* |
| MOL005320 | arachidonate | 45.57 | 0.2 | *Panax ginseng C. A. Mey.* |
| MOL005321 | Frutinone A | 65.9 | 0.34 | *Panax ginseng C. A. Mey.* |
| MOL005344 | ginsenoside rh2 | 36.32 | 0.56 | *Panax ginseng C. A. Mey.* |
| MOL005348 | Ginsenoside-Rh4_qt | 31.11 | 0.78 | *Panax ginseng C. A. Mey.* |
| MOL005356 | Girinimbin | 61.22 | 0.31 | *Panax ginseng C. A. Mey.* |
| MOL005357 | Gomisin B | 31.99 | 0.83 | *Panax ginseng C. A. Mey.* |
| MOL005360 | malkangunin | 57.71 | 0.63 | *Panax ginseng C. A. Mey.* |
| MOL005360 | malkangunin | 57.71 | 0.63 | *Jujubae Fructus* |
| MOL005376 | Panaxadiol | 33.09 | 0.79 | *Panax ginseng C. A. Mey.* |
| MOL005384 | suchilactone | 57.52 | 0.56 | *Panax ginseng C. A. Mey.* |
| MOL005399 | alexandrin_qt | 36.91 | 0.75 | *Panax ginseng C. A. Mey.* |
| MOL005401 | ginsenoside Rg5_qt | 39.56 | 0.79 | *Panax ginseng C. A. Mey.* |
| MOL006129 | 6-methylgingediacetate2 | 48.73 | 0.32 | *Zingiber officinale Roscoe* |
| MOL006936 | 10,13-eicosadienoic | 39.99 | 0.2 | *Arum Ternatum Thunb* |
| MOL006937 | 12,13-epoxy-9-hydroxynonadeca-7,10-dienoic acid | 42.15 | 0.24 | *Arum Ternatum Thunb* |
| MOL006957 | (3S,6S)-3-(benzyl)-6-(4-hydroxybenzyl)piperazine-2,5-quinone | 46.89 | 0.27 | *Arum Ternatum Thunb* |
| MOL006967 | beta-D-Ribofuranoside, xanthine-9 | 44.72 | 0.21 | *Arum Ternatum Thunb* |
| MOL007213 | Nuciferin | 34.43 | 0.4 | *Jujubae Fructus* |
| MOL008034 | 21302-79-4 | 73.52 | 0.77 | *Jujubae Fructus* |
| MOL008206 | Moslosooflavone | 44.09 | 0.25 | *Scutellariae Radix* |
| MOL008647 | Moupinamide | 86.71 | 0.26 | *Jujubae Fructus* |
| MOL008698 | Dihydrocapsaicin | 47.07 | 0.19 | *Zingiber officinale Roscoe* |
| MOL010415 | 11,13-Eicosadienoic acid, methyl ester | 39.28 | 0.23 | *Scutellariae Radix* |
| MOL012245 | 5,7,4'-trihydroxy-6-methoxyflavanone | 36.63 | 0.27 | *Scutellariae Radix* |
| MOL012246 | 5,7,4'-trihydroxy-8-methoxyflavanone | 74.24 | 0.26 | *Scutellariae Radix* |
| MOL012266 | rivularin | 37.94 | 0.37 | *Scutellariae Radix* |
| MOL012921 | stepharine | 31.55 | 0.33 | *Jujubae Fructus* |
| MOL012940 | Spiradine A | 113.52 | 0.61 | *Jujubae Fructus* |
| MOL012946 | zizyphus saponin I_qt | 32.69 | 0.62 | *Jujubae Fructus* |
| MOL012961 | jujuboside A_qt | 36.67 | 0.62 | *Jujubae Fructus* |
| MOL012976 | coumestrol | 32.49 | 0.34 | *Jujubae Fructus* |
| MOL012980 | Daechuine S6 | 46.48 | 0.79 | *Jujubae Fructus* |
| MOL012981 | Daechuine S7 | 44.82 | 0.83 | *Jujubae Fructus* |
| MOL012986 | Jujubasaponin V_qt | 36.99 | 0.63 | *Jujubae Fructus* |
| MOL012989 | Jujuboside C_qt | 40.26 | 0.62 | *Jujubae Fructus* |
| MOL012992 | Mauritine D | 89.13 | 0.45 | *Jujubae Fructus* |
| MOL013187 | Cubebin | 57.13 | 0.64 | *Radix Bupleuri* |
| MOL013357 | (3S,6R,8S,9S,10R,13R,14S,17R)-17-[(1R,4R)-4-ethyl-1,5-dimethylhexyl]-10,13-dimethyl-2,3,6,7,8,9,11,12,14,15,16,17-dodecahydro-1H-cyclopenta[a]phenanthrene-3,6-diol | 34.37 | 0.78 | *Jujubae Fructus* |
